# A Low-Cost Multi-Sensor Data Acquisition System for Fault Detection in Fused Deposition Modelling

**DOI:** 10.3390/s22020517

**Published:** 2022-01-10

**Authors:** Satish Kumar, Tushar Kolekar, Shruti Patil, Arunkumar Bongale, Ketan Kotecha, Atef Zaguia, Chander Prakash

**Affiliations:** 1 Symbiosis Institute of Technology, Symbiosis International (Deemed University), Pune 412115, India; tusharkolekar24@gmail.com (T.K.); shruti.patil@sitpune.edu.in (S.P.); arun.bongale@sitpune.edu.in (A.B.); 2 Symbiosis Centre for Applied Artificial Intelligence, Symbiosis International (Deemed University), Pune 412115, India; 3 Department of Computer Science, College of Computers and Information Technology, Taif University, P.O. Box 11099, Taif 21944, Saudi Arabia; zaguia.atef@tu.edu.sa; 4 School of Mechanical Engineering, Lovely Professional University, Jalandhar 144411, India; chander.mechengg@gmail.com

**Keywords:** Arduino, data acquisition system, fault detection, fused deposition modelling, low-cost, multi-sensor

## Abstract

Fused deposition modelling (FDM)-based 3D printing is a trending technology in the era of Industry 4.0 that manufactures products in layer-by-layer form. It shows remarkable benefits such as rapid prototyping, cost-effectiveness, flexibility, and a sustainable manufacturing approach. Along with such advantages, a few defects occur in FDM products during the printing stage. Diagnosing defects occurring during 3D printing is a challenging task. Proper data acquisition and monitoring systems need to be developed for effective fault diagnosis. In this paper, the authors proposed a low-cost multi-sensor data acquisition system (DAQ) for detecting various faults in 3D printed products. The data acquisition system was developed using an Arduino micro-controller that collects real-time multi-sensor signals using vibration, current, and sound sensors. The different types of fault conditions are referred to introduce various defects in 3D products to analyze the effect of the fault conditions on the captured sensor data. Time and frequency domain analyses were performed on captured data to create feature vectors by selecting the chi-square method, and the most significant features were selected to train the CNN model. The K-means cluster algorithm was used for data clustering purposes, and the bell curve or normal distribution curve was used to define individual sensor threshold values under normal conditions. The CNN model was used to classify the normal and fault condition data, which gave an accuracy of around 94%, by evaluating the model performance based on recall, precision, and F1 score.

## 1. Introduction

The additive manufacturing process [1] is a growing manufacturing field that uses layer by layer deposition of the material to produce a finished product [2]. Additive manufacturing (AM) uses CAD/CAM software to generate 3D products that are further converted into G-codes using slicer software. The G-codes file is provided to the 3D printer for producing 3D printed objects. It performs layer-by-layer deposition of material and follows a G-codes sequence to print an object [1]. Along with G-codes, many AM processes use their own vector generation mechanism [3]. Digital light processing (DLP), fused deposition modelling (FDM) [4,5,6], selective laser sintering (SLS), and stereolithography (SLA) [7] are the types of AM processes. The FDM-based 3D printer is popular for producing low-cost and complex geometrical objects while reducing the printing time. Along with several benefits, FDM also leads to defects in final printed products due to various reasons. The faults occur in objects resulting from changing the process parameters, such as disturbed bed leveling, changes in nozzle or extruder temperature, structural looseness, etc. [5].

The majority of defects arises due to changes in temperature and vibration caused during the printing stage. Due to a material property or printing process failure, the FDM 3D printed component is produced with a variety of defects, including warping, poor surface finish, porosity (infill gap), stringing, cracking, and so on [1,8]. The author in [9] mentioned the various 3D printing defects and provided a possible solution to reduce those defects. A few other methods, such as X-ray computed tomography (XCT) and active infrared thermography (IRT), can also be used to quantify defects in the additive manufacturing process [10,11]. The warping defect in the 3D object occurs when the material deposition is non-uniform, and there is a temperature difference between the nozzle and bed during the printing process. Another defect caused due to solidification is porosity. Porosity is the initial stage of producing cracks in the finished product. It appears due to entrapped gas bubbles, lack of fusion, solidification shrinkage, and loss of material properties due to humidity and moisture [12]. Defects also occur in the product with the increasing speed of cooling or heating due to the generation of shear stresses. To deliver quality products in the market, detecting these defects in FDM printing is crucial. Diagnosing defects that occur during 3D printing is a challenging task. Proper data acquisition and monitoring systems need to be developed for effective fault diagnosis. Proper classification of faulty products can also help in finding the causes of faults. In this work, the authors aim to detect and classify the different types of defects that occur in FDM-based 3D printing. 

## 2. Related Work

Proper fault diagnosis is an essential aspect in additive manufacturing for producing good quality products. The faults can be diagnosis by using data-driven methods [13]. Cyber-physical manufacturing [14] is an emerging area of the manufacturing process. It deals with physical systems and interacts with the cyber domain through a communication network [15]. In the cyber domain, physical system process parameters can be monitored through each clock cycle of the communicating network using a sensing element. The various employed sensing elements on the physical system can measure multiple process parameters such as vibration, magnetic field, current, acoustic, temperature, humidity, etc. Based on real-time sensor data, the anomaly can find out and represent an actual physical system in the cyber domain to accurately predict the behavior of the physical system [16]. The actual physical system interacts with humans and environmental parameters that change the physical system’s state, which is one of the significant issues in the physical domain [17]. High-end FDM can monitor, but low-cost FDM does not have process monitoring capabilities. In such a case, real-time data capturing and monitoring is possible using an external data acquisition system and using multiple sensors. Even though the high-end FDM machines have monitoring capabilities, they do not provide the provision to classify the normal and abnormal (faulty) data for the analytics. Proper data analytics helps to classify the causes of fault as well. Kousiatza et al. used thermocouple and fiber Bragg grating (FBG) sensors to measure real-time field parameters and their effects on multilayer deposition to identify anomalies [4]. In the additive manufacturing process, the data-driven with predictive approach [18] is used to improve the surface integrity of the finished product. Using multiple sensors such as infrared temperature, thermocouples, and accelerometer, real-time temperature and vibration data were captured, and using machine learning techniques, the quality of surface roughness of the finished product was measured [19].

### 2.1. Monitoring Models Used in AM Process

Deep neural networks (DNNs) [20] with convolutional neural networks (CNNs) are used to employ an automatic quality gradient system in AM processes. By capturing real-time layer-by-layer deposition, the internal and surface defects in the finished object in 3D printing can be measured and classified into different types [21]. When large objects are printed using an FDM-based 3D printer, most of the defects occur between layers of the printing process due to misalignment, leading to wastage of material and time. To prevent wastage of material caused due to misalignment of objects, mechanical components and structures in the AM process are introduced with a computer vision system. Langeland et al. used two cameras set at different angles to capture real-time material deposition images to train a deep learning model that further predicted defects based on the surface [22]. The quality of 3D product maintenance is a big challenge in AM processes. However, predicting defects in the 3D product is possible using image processing and supervised machine learning models. Based on support vector machine models, datasets are classified into two parts: abnormal and normal image datasets. Delli et al. performed an experiment on acrylonitrile butadiene styrene (ABS) and polylactic acid (PLA) materials, and based on their surface finishing, the part geometry types of defects introduced in the finished objects were predicted [23]. Stoyanov et al. found that the machine learning models referred to the predictive process control system to improve product quality and investigated ink-jet electronics devices using a data-driven method [24]. In the stereolithography 3D printer device, a high level of concentrated oxygen is used. Monitoring real-time oxygen levels during the printing process is possible using the data-driven method, which measures the normal and critical level oxygen levels to prevent a high risk of quality degradation [7].

### 2.2. Low-Cost Data Acquisition Systems

González et al. developed a low-cost Arduino-based DAQ system for automobile dynamics applications [25]. For validation of a low-cost DAQ system, it is compared with standard vibration acquisition technologies. Designed low-cost DAQ shows an error of 2.19% compared to the standard vibration acquisition system. Gopalakrishna et al. used an accelerometer, and a low-cost acquisition system was built to monitor a bearing in a mechanical system and relay the data to the end devices [26]. Shen et al. used an open-source Arduino micro-controller for the behavior monitoring of mussels [27]. The mussels’ behavioral responses were observed and analyzed. Mussels’ heart activities and valve motions were tracked using infrared and Hall sensors. During the exposure, mussels displayed a typical behavior pattern, which was captured by the low-cost Arduino microcontroller board. Barile et al. developed a real-time automated process to assess the structural integrity of dynamic events by transmitting data such as temperature, position, acceleration, and inclination wirelessly via the system’s harvested energy [28]. Titov et al. developed a new design of the CaPaMan with low-cost, lightweight characteristics using a 3D printer [29]. The effectiveness of the design circuit was tracked with the help of an Arduino controller and sensors. Damcı et al. developed a low-cost acquisition system designed for a single-axis vibration shaker table using an Arduino DUE board [30]. Paladino et al. developed a low-cost monitoring system in the food processing industry to control the quality of food products using the free, open-source kernel low-cost supervisory control and data acquisition (SCADA) system [31]. Soto-Ocampo et al. developed a low-cost and high-frequency DAQ system using a Raspberry Pi microcontroller to diagnose a bearing test rig [32]. Vidal-Pardo et al. developed a five-channel Arduino-based DAQ system for aerodynamic applications [33]. The literature found that developing such a low-cost DAQ system has a considerable amount of application areas.

Many researchers use the Arduino and Raspberry Pi-based microcontroller to develop low-cost DAQ systems to diagnose the different types of faults in 3D printers and other machines [34,35]. The low-cost DAQ system has limitations of sampling frequency. The traditional DAQ system with a single sensor may not capture all types of defects in the AM process. The single sensor has a few drawbacks, as explained in Sayyad et al. [13], so the multi-sensor approach is preferred for reliable results. During AM printing, the change in process temperature and vibrations are essential monitoring parameters. These parameters can only be monitored by using a multi-sensor approach. Changes in temperature can easily be detected by temperature sensors, whereas vibrations generated due to structural defects can easily be captured using vibration sensors only. Therefore, the use of multiple sensors is necessary in AM process fault detection. However, there has not been much research addressing multi-sensor low-cost DAQ systems. The analysis of acquired data using feature extraction is also missing in most of the literature.

In this paper, the authors focused on the following points:

1. The authors proposed a low-cost multi-sensor data acquisition system for the 3D printer to detect various faults induced in the FDM 3D printed products during the printing process.
2. In this work, the authors considered the different fault conditions for 3D printers during experimentation, such as disturbing bed leveling, changing nozzle temperature, changing belt tension, etc., and inducing severe defects in the finished product.
3. The data acquired by the designed DAQ system refer to performing time and frequency domain analysis to extract various features from it and perform multiple fault diagnoses using the CNN model.

## 3. Methodology

**Figure 1:**
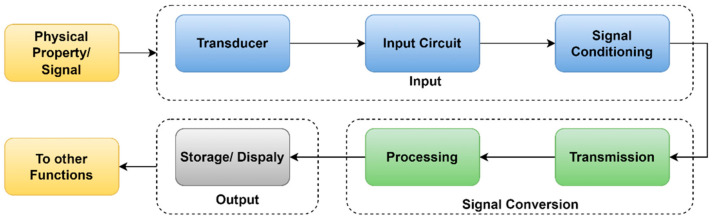
The basic architecture of DAQ.

The data acquisition system is used to measure and record multiple sensor data, which helps to analyze various faults and their conditions. The design of DAQ for dynamically complex systems for low-cost platforms confronts various challenges, including a data retrieval rate constraint [36]. In this proposed system, the DAQ is designed to capture multi-sensor data through a different channel with the same sampling frequency. This DAQ is used to measure vibration, sound, and current sensor output, which is mounted on the 3D printer. The basic architecture of the data acquisition system is shown in Figure 1.

### 3.1. Data Acquisition and System Design

The essential components of DAQ are the input device, signal conditioning unit, data preprocessing unit, and output device. Input devices deal with sensors that measure real-world or physical parameters such as vibration, humidity, temperature, and sounds that convert signals into electrical form [37]. In the second step, the signal is amplified and converted into a microcontroller readable format by using voltage to frequency converter, analog to digital converter, and digital to analog converter circuits. In the third step, raw data are processed and converted into their measuring unit by performing mathematical operations. In the fourth step, generated data from the sensor are given to the output device for data monitoring and storing purposes.

This proposed system uses vibration, current, and sound sensors as the input signals. VBR1/D0-3 is an industrial vibration sensor that is connected to the 3D printer and transmits data to an Arduino using the RS485 module. The Max4466 adjustable gain sound sensor is used to measure the sound intensity. It is connected to the analog to digital converter of the Arduino board to measure sensor output. The ADS1015 operational amplifier is used to amplify the current sensor output and is sent to the Arduino using Inter-Integrated Circuit, eye-squared-C communication (I2C). Through different terminals of the Arduino Uno, different sensor data are measured by keeping a constant sampling frequency. The detailed specification of the Arduino Uno, power supply, sensors, Analog to digital converter (ADS1015), and RS485 Module is added in Appendix A.

**Figure 2:**
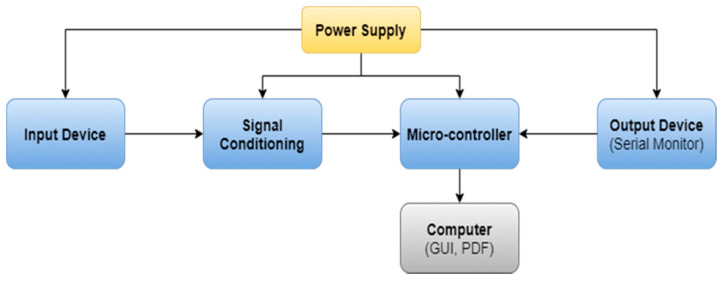
Architecture of DAQ.

In this data acquisition system, multi-sensor data were monitored and recorded with design GUI and Python IDE (Integrated Development Environment) software and stored in pdf format with a sampling frequency of 140 Hz. Figure 2 shows the DAQ architecture layout. It is a prototype of the existing system designed and simulated from Proteus software to check system feasibility.

**Figure 3:**
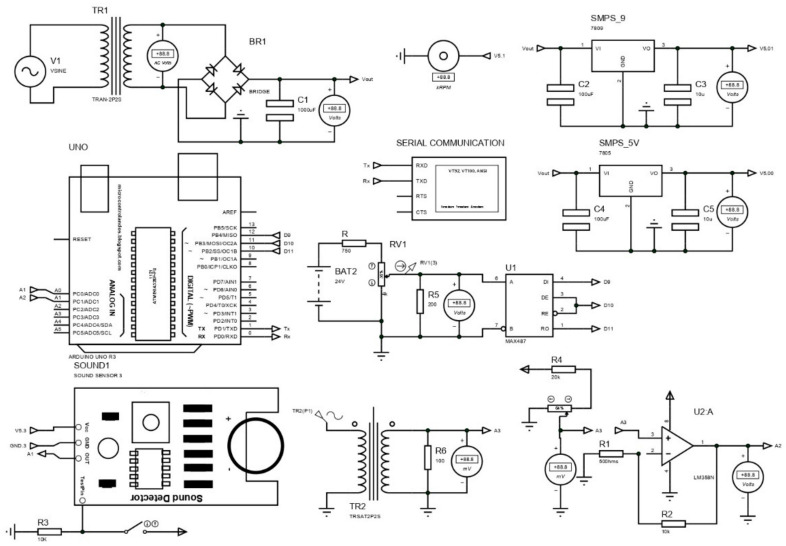
Proposed system PCB layout in Proteus software.

The components used in Figure 3 are the current transformer, sound sensor, step-down transformer, Max487 IC, voltage regulator IC (7805, 7809), cooling fan, an Arduino Uno board, etc. The 230 AC supply is given to a step-down transformer that gives 12.9 AC output that further convert into 15.6 DC volts using a full-wave bridge rectifier circuit. For 5 V and 9 V supplies, voltage regulators IC 7805 and 7809, respectively, are used. Here, the voltage drop across the IC generates heat energy that must be dissipated from the design printed circuit board (PCB). Otherwise, it affects the performance of the hardware. This heating problem was solved using a cooling fan with a mass flow rate of 60 cubic feet per minute (CFM), as explained in Appendix B. As shown in Figure 3, the voltage divider circuit is connected to R5 resistance in parallel. It gives output that varies from 4 to 20 mA, such as the output provided by the analog terminal of the vibration sensor. The current transformer is used to measure changing current by connecting burden resistance across it. It gives analog output that must be amplified before being provided to the Arduino board. The LM358 op-amp is used to amplify the current transformer output that is further sent to the Arduino board to measure a change in current.

**Figure 4:**
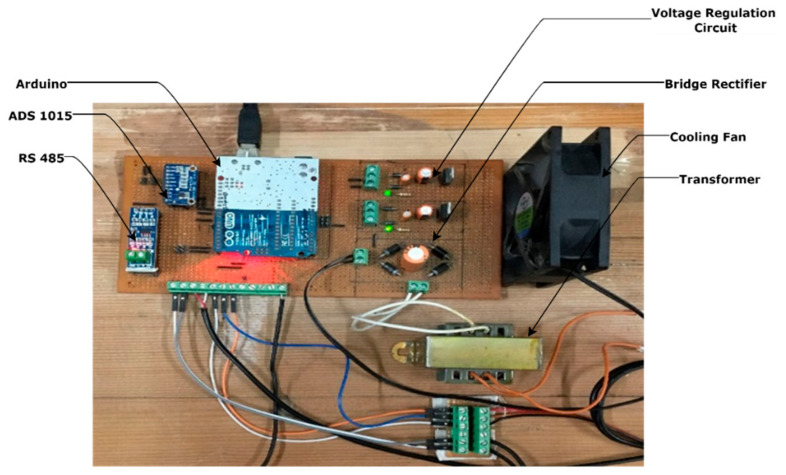
The final layout of PCB.

Here a max4466 adjustable gain sound sensor is used that offers analog output as per changing sound intensity. Thus, no external amplification circuit is required for it. Using the analog terminal of the vibration sensor, only resultant vibration is measured, but by using the RS485 communication terminal, the individual axis of the vibration is measured, which is more effective to troubleshoot faults induced in the 3D printed products. Thus, the RS485 module is used to communicate with a vibration sensor and sends the individual axis data to the Arduino board. Here current sensor analog output is given to a design op-amp that provides better output for high ampere current, but using an ADS1015 programable operation amplifier gives better output for low- or high-frequency signals. Therefore, the ADS1015 programmable amplifier was used to measure the output of the current sensor. The final layout of the circuit used for the proposed system is shown in Figure 4.

### 3.2. Location of Sensors

The sensor’s location mounted on the experimental setup decides the intensity of the variation measured by individual sensors. Most sensor locations affect the numerical value generated by individual sensors during various fault conditions. Therefore, a proper location for the sensor needs to be selected that captures effectively fault conditions and helps to improve diagnoses of various faults. In this proposed system, the design DAQ is connected to sensors mounted on the FDM to measure faults induced during the printing process. In a 3D printer, stepper motors are used, which are some of the major components responsible for generating vibration, noise, and changing currents and electromagnetic fields. Different sensors are mounted on a 3D printer based on the stepper motor position.

**Figure 5:**
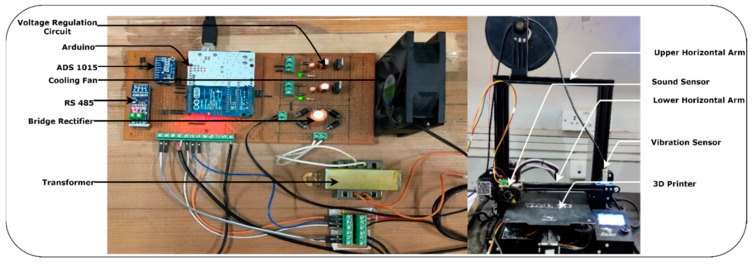
Experimental setup for the proposed system.

**Figure 6:**
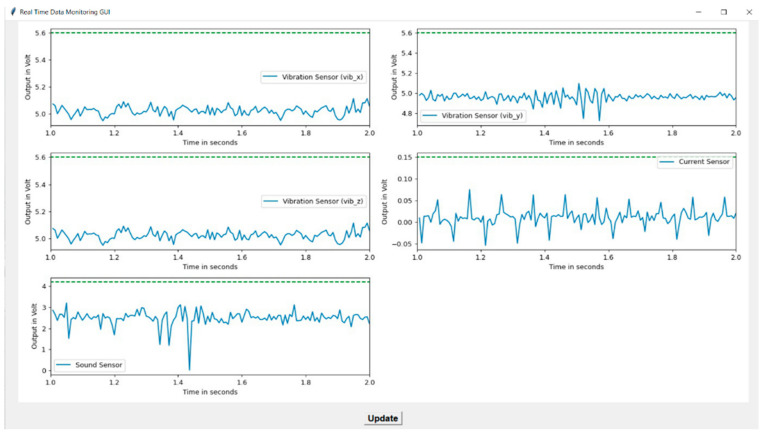
Graphical user interface (GUI) for DAQ.

Figure 5 shows the experimental setup; on the lower horizontal beam-like structure, the vibration sensor is mounted because two stepper motors perform their individual operations and produce the vibration that is clearly measured in the lower horizontal beam compared to the upper horizontal beam. This attached vibration sensor is used to measure the three axes of vibration. The mounted sound sensor measures the noise produced by the stepper motor. It is placed near the nozzle and the third stepper motor, which is always closer to the deposited material layer to produce the 3D printed product or object. It captures noise or sound during printing operations that help to analyze faults induced in 3D products. The 3D printer requires a constant power supply to print an object, but printing or environmental conditions cause fluctuations in the ability of the supply current to keep a constant supply voltage. Clamp-type current transformers are used to measure fluctuations in current, which are caused due to factors such as printing speed, nozzle temperature, belt tension, disturbed bed leveling, etc. It is clamped to the live wire of the single-phase supply in order to measure fluctuations in current. It is placed near to the DAQ system that measures real-time sensor data with a timestamp. A separate graphical user interface (GUI) was developed using the Python programming language. This GUI records and displays real-time sensor data in a graphical format, as shown in Figure 6. It represents a real-time data monitoring GUI that shows the real-time status of the 3D printer during the printing process. The multiple sensors are mounted on the 3D printer to monitor the health conditions of 3D printed products and capture variations in signals with fault conditions. The GUI represents vibration, current, and sound sensor signals. The *y*-axis represents variation in signals in terms of voltage, whereas the *x*-axis represents the time duration for which signals are captured. When a fault occurs in the finished product, it gets replicates in the form of variations in signals that are anomalies, as shown in Figure 6. The green dotted line shown in the GUI is used to classify the signal into normal and abnormal forms using the threshold value. Threshold values are calculated from the probability distribution plot based on the maximum number of data points lying in normal printing conditions. The detailed procedure for anomaly detection is explained in Section 4.6.

### 3.3. Experimental Conditions

After completing the hardware and software part, a designed DAQ was used to analyze the fault induced in 3D printed products. These captured multisensor signals were used for anomaly detection using a clustering algorithm. Mainly in the 3D printer, the fault is induced in the product due to mechanical or electrical system failure. In mechanical failure, the fault is induced mechanically, which has a major contribution with parameters such as disturbed bed leveling, a loose assembly of the stepper motor, frame, nozzle, belt tension, change in bed, and nozzle temperature, feed rate, etc. In electrical failure, hardware and power supply failure are major contributors. Some of the faults occur due to wrong information fed through slicer software. The authors induced defects in 3D printing and predicted reasons responsible for generating faults in the final product. Overheating, warping, messiness, elephant foot, infill gap, level shift, z-wobble or side layer issue, cracking, and stringing are such faults that are mapped with their cause of fault generation, such as 180 °C temp, 260 °C temp, high belt tension, low belt tension, low bed level and bed level high, etc., as described in Table 1. A simple cube of the 30 mm × 30 mm × 20 mm dimension was printed to capture the effects of the fault condition caused in the 3D product.

The different faults generated during the printing are listed below.

Warping: In FDM-based 3D printed products, the bottom surface is bent in the upward direction to cause a warping defect. It mostly occurs with insufficient adhesion between extrusion plastic with the print surface, and thermal contraction of the surface [38].

Cracking (delamination): In the 3D printing process, two-layers cannot stick together properly and cause the separation of layers; such an effect is known as delamination or cracking. It occurs due to poor adhesion and thermal shrinkages [38,39].

Layer Shifting: In the printing process, there is a certain displacement of the axis that occurs up to a certain height due to mechanical failure or lack of power given to motors, causing overheating of the motor, which loses its functionality [9].

Z-wobble or side layer issue: This is the most common defect introduced in 3D products and forms an inconsistency in wall alignment. Such types of defects mostly occur due to structural failure and excessive tightening of *z*-axis motion affecting components [40].

Overheating or curling: This type of defect occurs when material melts at too high a temperature, and there is not enough time to cool material before forming layer by layer deposition [40].

Elephant foot: Expansion of the first layer during printing introduces elephant foot defects in the 3D product. These faults occur because the nozzle is too close to the bed surface [38].

Infill Gap: It occurs in 3D printed object surfaces due to changing printing speed or a slightly blocked surface nozzle, resulting in a disturbed quality of the finish product [38].

**Table 1:** Fault condition and fault images with labels.

Normal and Induced Fault Condition	Description	FDM Product	Faults
Normal Condition	Bed and extrusion temperatures are kept at 50 °C and 200 °C throughout the printing process. The bed levelling is also uniform, using an adjustable screw connected to the levelling mechanism on which the 3D printer bed is mounted. The link connected to the horizontal beam and pully is tight enough to prevent the belt from slipping.	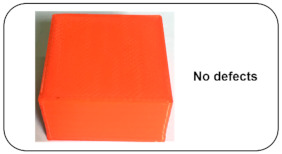	No fault
Disturbed Bed Leveling (Level up)	Bed and extrusion temperatures are kept at 50 °C and 200 °C, respectively, throughout the printing process, and all links connected to horizontal columns are tight enough to prevent slippage. The bed leveling is disturbed using the adjustable screw that blocks the nozzle from the front side.	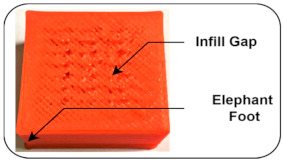	Poor infill [39,41] Elephant foot [38]
Disturbed Bed Leveling (Level down)	Bed and extrusion temperatures are kept at 50 °C and 200 °C, respectively, throughout the printing process, and all links connected to horizontal columns are tight enough to prevent slippage. The bed leveling is disturbed using the adjustable screw that keeps the distance between the nozzle and bed surface approximately 0.2 cm that prints the first few layers in the air.	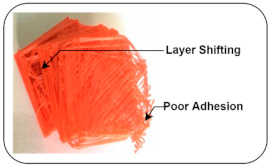	Layer shift [9] Poor adhesion [9]
Disturbed Extrusion Temperature: 260 °C	The bed level is uniform, and all links are tight enough to avoid slippage. The temperatures of the nozzle and bed are kept at 260 °C and 50 °C, respectively. Due to increasing nozzle temperature, the quality of the finished surface gets reduced.	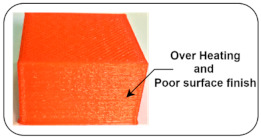	Overheating [40] Poor surface finish [40]
Disturbed Extrusion Temperature: 180 °C	The bed level is uniform, and all links are tight enough to avoid slippage. The nozzle and bed temperature are kept at 180 °C and 50 °C, respectively, due to decreasing nozzle temperature and causing weak layer deposition. This leads to cracking and edge warping defects in the final product.	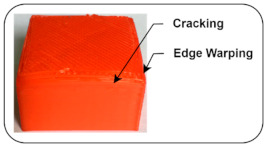	Cracking or layer separation [38,39] Edge warping [38]
Low Belt Tension	The bed and nozzle temperatures are kept at 50 °C and 200 °C, respectively, with uniform bed leveling. The link that connects the horizontal beams to the pully, which helps the stepper motor drive the nozzle assembly along a horizontal axis, becomes loosened.	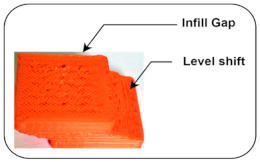	Infill gap [41] Level Shift [38]
High Belt Tension	The bed and nozzle temperature are kept at 50 °C and 200 °C, respectively, with uniform bed leveling. The link that connects horizontal beams to the pully, which helps the stepper motor drive the nozzle assembly along a horizontal axis, is tight enough.	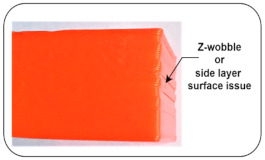	Z-wobble or side layer surface issue [40]

### 3.4. Data-Driven Model

**Figure 7:**
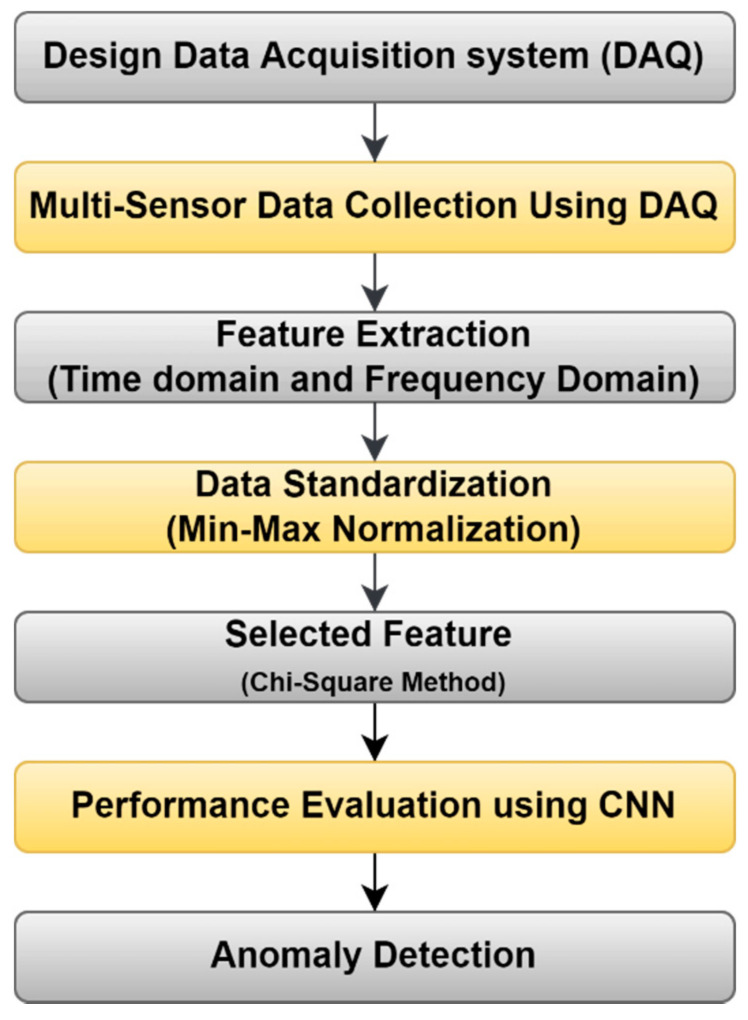
Flow chart for the data-driven model for anomaly (fault) detection.

The data-driven model mainly deals with the collection of the raw signal data from the different sensors, standardization of raw signals, and extracting and selecting the most significant features to train machine learning or deep learning models. The flow of the data-driven model is shown in Figure 7.

In this proposed method, multiple sensor data were collected from the design DAQ to prepare the dataset. Time and frequency domain analysis was performed on a raw signal to extract various features that further form a feature vector. The number of feature counts was reduced by using the chi-squared method. It selected the most significant features based on the chi score and fed them to the CNN model to perform multiclass classification. The trained model performance was evaluated using a confusion matrix based on performance evaluation parameters such as precision, F1-score, and recall. After classification, k-means cluster algorithms were used to generate two clusters fed to calculate the normal distribution curve to find out lower and upper threshold values for multi-sensor data of the normal condition. Based on a threshold value, anomalies from multiple sensor data were detected.

## 4. Results and Discussion

The result section describes the multi-sensor data analysis, feature selection, feature selection, and model performance evaluation using the CNN model. The power supply unit and sensor configuration part are described in Appendix B.

### 4.1. Multi-Sensor Data Analysis

Evaluating the performance of individual channels used for collecting data is achieved by observing a maximum number of samples recorded by each channel within one second. The sampling frequency of the individual channel is calculated by dividing the maximum sampling frequency of the device by the number of channels. The sampling frequency is decreased with an increase in the number of channels. In this proposed system, four-channels or pins of the Arduino were used to measure the current, sound, and vibration data. I2C and RS485 communication were used for current and vibration sensors to capture real-time data. Through serial real-time data monitoring of three individual axes of vibration, the current and sound sensor monitored at a baud rate of 115,200. The device port and baud rate were configured in Python using the serial library and storing Arduino serial data in pdf format. Various combinations of sensors and their effects on sampling frequency were measured and are described in Table 2.

In this proposed work, a simple cube object is printed with the 3D printer during normal conditions. At the same time, real-time data are captured using multiple sensors. Then, different faults are induced in the printing process manually Table 1, and faulty data are captured.

**Figure 8:**
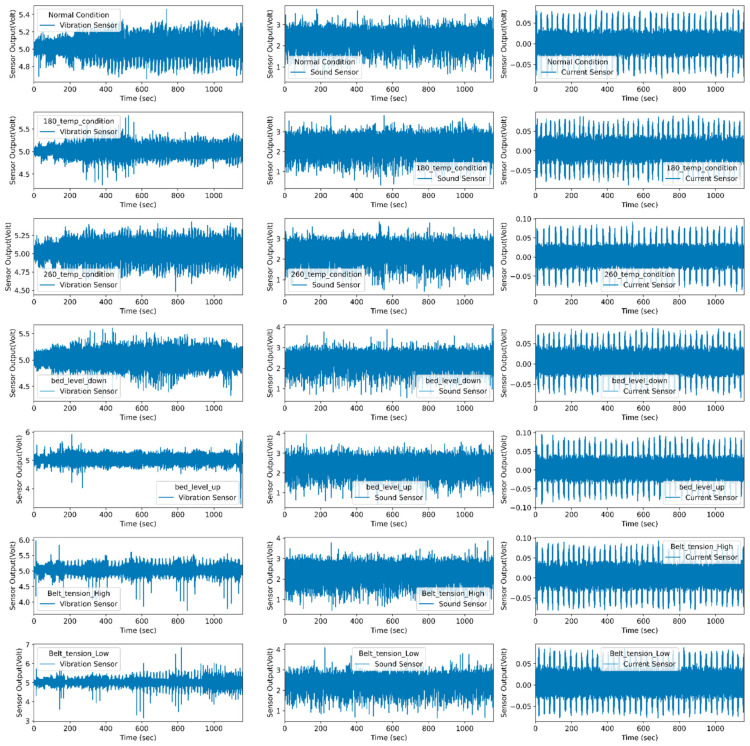
Raw sensor signal representation for normal and fault conditions.

This proposed method uses vibration, sound, and current sensors for data collection purposes. Multisensor data efficiently capture the various fault condition and their corresponding effect. Vibration sensors capture the change in vibration intensity during the printing stage. They can easily detect the structural defects occurring in FDM. Using the current and sound sensor, the health condition of the 3D printer is easy to diagnose. The current sensor is used to measure the fluctuation in the power supply. As faults are induced in the machine, the current drawn by the stepper motors varies, and the corresponding noise generated by the individual motor is also changed. The sound sensor is used to capture the noise produced by the individual motors under normal and abnormal conditions. Figure 8 shows the raw signal representation for the normal and abnormal conditions.

### 4.2. Feature Extraction

The raw data collected from the sensor has large dimensions. Processing such high-dimensional data may require more computational resources and time. Feature extraction is helpful to extract the most significant features from the raw data for analysis. These features can be extracted in time and frequency domains. This paper considers the time-domain features such as mean, root mean square, peak to peak, variance, skewness, shape factor, clearance factor, etc., and from the frequency domain, considering features such as spectral mean, spectral variance, spectral standard deviation, spectral skewness, etc., to extract time and frequency domain features. Table 3 shows the formulae used for extracting different features. The same formulas calculate time and frequency domain features for each sensor signal and create a feature vector.

### 4.3. Data Normalization/Standardization

The extracted time and frequency domain have different numerical values. It is also possible that the low dimensional feature should have a good correlation with predicted output, and due to the high dimension of the feature, it may dominate the performance of the low dimension feature. This problem is solved by referring to scaled data using data normalization methods. In this proposed method, min–max methods are referred for data normalization with a 0 to 1 scaling range. The extracted features are scaled using a min–max scaler, and their mathematical form is represented in Equation (1) [42].

(1) $$z_{\min\_max}=\frac{y_{i}-\min\left(y\right)}{max\left(y\right)-\min\left(y\right)}$$

### 4.4. Feature Selection Using the Chi-Square Method

The chi-square method is used to select the relevant features from extracted time and frequency domains. The selected features are provided to classification algorithms for performing multi-fault classification. In this paper, the chi-square method is used for feature selection purposes. This method is generally used for checking the independence of two variables. Equation (2) is used for calculating the chi-square values [43]:

(2) $$X^{2}{}_{d}=\sum \frac{{\left(X_{i}-Y_{i}\right)}^{2}}{Y_{i}}$$

where *d* = degrees of freedom, *X* = observed values, and *Y* = expected values.

**Figure 9:**
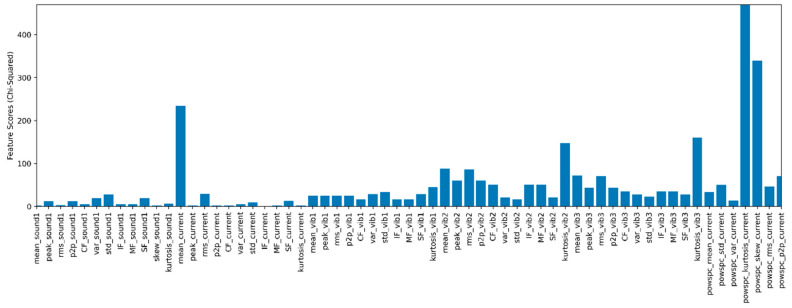
Feature selection using the chi-square method.

When the observed values are close to the expected values, the value of chi-square is low. The low chi-square value indicates the variables have high independence, i.e., low dependency. Thus, for high correlation or dependency, high chi-square values are selected in the feature selection process. Figure 9 represents the time and frequency domain feature nature evaluated by the chi-square method. The optimum number of features is selected from this graph by defining the threshold value. This threshold value is randomly selected, and the corresponding feature count is fed to the CNN model to evaluate model performance. The author refers to time and frequency domain features calculated from Table 1 to create a feature vector. As the number of the feature count increases, it increases computation time. The most significant and relevant features are fed to the CNN model to reduce computational time and avoid overfitting problems.

Each feature has different dimensions and numerical values that affect model performance and induce the effect of the curse of dimensionality. Therefore, selecting the most relevant features from the generated feature vector can reduce the computation cost and improve classification accuracy. Table 4 represents selected features based on their chi-square value and describes the multiple sensor feature contributions with the predicted value.

### 4.5. Model Performance Evaluation Using CNN

This section mainly discussed the working of the CNN model, performance evaluation of the CNN model, learning curves for CNN model, and multi-fault diagnosis using the CNN model.

#### 4.5.1. Working of CNN Model

**Figure 10:**
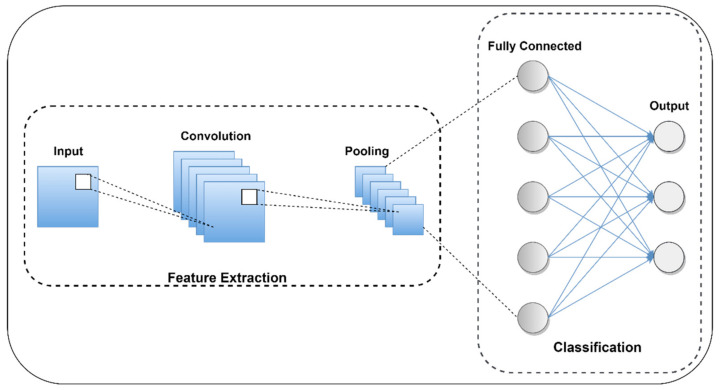
Basic architecture of the CNN model.

CNN is a conventional neural network mainly used for multi-class image classification, diagnosis, and prognostic fields due to its feature mapping and data mining capability [44]. Figure 10 represents the architecture of the CNN model [45,46]. It automatically extracts the most significant features from different input data using various kernel filters. To obtain high-performance output from different algorithms, mostly preprocess data are used but using the CNN model without preprocessing data may give high-performance output. The basic structure of CNN is a composite of the input layer, alternating convolution layer, and pooling layer with equal padding that could be converted into low dimension using a flatten layer to give the desired output by referring to a dense layer [44]. In between the convolution layer with equal padding is mostly used relu as an activation function that replaces the negative value by zero. The output predicting layers of CNN are mostly composed of sigmoid activation functions that scale feed data through the range of 0 to 1. The selected features from the time and frequency domain are fed to the CNN input layer to perform multi-class fault classification. It performs feature extraction and classification simultaneously using a neural network. It includes three convolutional layers, three pooling layers, and a single dropout layer used to connect the fully connected layer.

##### Convolution Layer

The forward propagation process uses kernel filters and activation functions to perform convolution operations. A feature vector made by a feature matrix is obtained in the convolution layer that performs feature mapping shown in Equation (3) [47]:

(3) $$x_{j\;}^{l}=f\left(\sum_{i=m_{j}^{l}}\left(\;x_{j}^{l-1}+k_{ij}^{l}\right)+B_{j}^{l}\right)$$

where $x_{j\;}^{l}$ is *l*th layer map with a *j*th feature, *f* is the activation function, $m_{j}^{l}$ is a group of the input to be calculated by output, $k_{ij}^{l}$ is the kernel used for lth layer, and $B_{j}^{l}$ is the bias of the lth layer.

In the backward propagation process, the back-propagation algorithm updates the convolutional layer, and its parameters or weight during every run of epochs to improve CNN model performance output are shown in Equation (4).

(4) $$\frac{\partial L}{\partial k_{ij}^{l}}=\sum_{a,b}\left({\left(\delta_{j}^{l}\right)}_{a,b\;}*\;{\left(p_{i}^{l-1}\right)}_{a,b}\right),\;\frac{\partial L}{\partial B_{j}^{l}}=\sum_{a,b}{\left(\delta_{j}^{l}\right)}_{a,b\;}\;$$

where $\left(p_{i}^{l-1}\right)$*_a_*_,*b*_ is the loss function that patches with $x_{j}^{l-1}$ and is multiplied element-by-element by $k_{ij}^{l+1}$ during convolution to compute the element at (,) in the output convolution result; $x_{j\;}^{l}$. $\delta_{j}^{l}$ is *j*th element of the sensitivities in the lth layer.

##### Pooling Layer

The number of layers connected to the convolutional layer of the CNN model can be reduced, but the number of neurons in each feature map is not reduced significantly, which leads to causing high dimension and overfitting of the model. To reduce the overfitting problem, a pooling layer is used. Equation (5) calculates the forward propagation of the pooling layer, and Equation (6) represents the back-propagation of the pooling layer.

(5) $$x_{j\;}^{l}=f\left(\beta_{j}^{l}\;*down\left(x_{j}^{l-1}\right)+B_{j}^{l}\right)$$

(6) $$\frac{\partial L}{\partial \beta_{j}^{l}}=\sum_{a,b}\left(\delta_{j}^{l}*down\;{\left(x_{i}^{l-1}\right)}_{a,b}\right)$$

Here, *down*(.) denotes a sub-sampling function, $\beta_{j}^{l}$ denotes multiplicative bias, and $B_{j}^{l}$ denotes the additive bias of the lth layers.

##### Fully Connected Layer

In this layer, all neurons are connected to each other, which means the previous layer neuron is connected to the next layer, as with multilayer perceptions, and the output from the fully connected layer is calculated by Equation (7).

(7) $$x^{l}=\alpha \left(w^{l}\;x^{l-1}+B^{l}\right)$$

where *B^l^* is the bias of the lth layer, *α*(.) is the activation function, and *w^l^* is the weight.

#### 4.5.2. Performance Evaluation Metrics

To evaluate the prediction performance of the classification problem, using machine learning or a deep learning model can be the preferred confusion matrix. It provides a confusion matrix report that tells us an individual class performance based on precision, recall, F1-score, and support. The output predicted by the CNN model is evaluated by the confusion matrix using *t_p_* (True Positive), *t_n_* (True Negative), *f_p_* (False Positive), or *f_n_* (False Negative) parameters. Precision represents the positive predictive value ratio calculated from the total positive prediction. It defines the number of positive cases that turn into positive values and is calculated by mathematical Equation (8).

(8) $$P_{precision\;}=\frac{t_{p}}{\;t_{p}+f_{p}}$$

where

*t_p_* is a true positive value, or actual and predicted value is true and positive.
*t_n_* is a true negative value, or actual and predicted value is true and negative.
*f_p_* is a false positive value, or the predicted output was a positive value, but the actual predicted output is negative.
*f_n_* is a false-negative value, or the predicted output was a negative value, but the actual predicted output is positive.

The recall shows how correctly our model predicts actual positive cases. It shows probability distribution and defines the sensitivity of the true predicted output, and its mathematical form is described in Equation (9).

(9) $$R_{recall\;}=\frac{t_{p}}{\;t_{p}+f_{n}}$$

Therefore, precision is essential in cases where false positive is higher than false-negative prediction values, and recall is necessary where false-negative is greater than false-positive predicted values. However, in actual implementation, the increased recall of the predicted output given model precision could be decreased and vice versa.

(10) $$F_{F1\_score}=\frac{2*P_{pricision}\;R_{recall}}{P_{pricision}+R_{recall}}$$

To solve such a problem, Equation (10) refers to and defines precision and recall in a single line using F1-sores. The F1-scores in the confusion matrix are given harmonic means recall and precision, and are maximal when both metrics parameters have the same or equal values.

#### 4.5.3. Learning Curves for CNN

**Figure 11:**
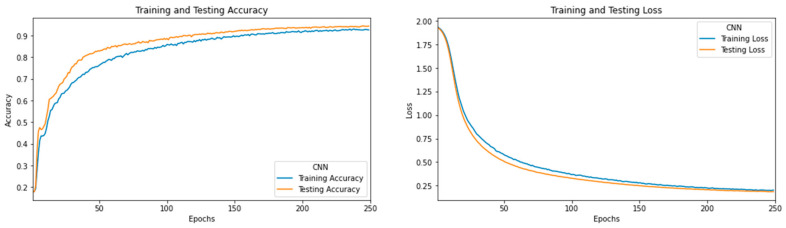
The learning curve of the CNN model for training and testing accuracy and losses.

The selected features from the chi-square method are fed to the CNN model to perform classification. For training the CNN model, 80% of data is considered, and the remaining 20% of the data are preferred for evaluating model performance. It gives 94% of testing accuracy on 20% of the test dataset, and corresponding accuracy and loss that occur during the model building process are shown in Figure 11.

#### 4.5.4. Multi-Fault Diagnosis Using CNN

**Figure 12:**
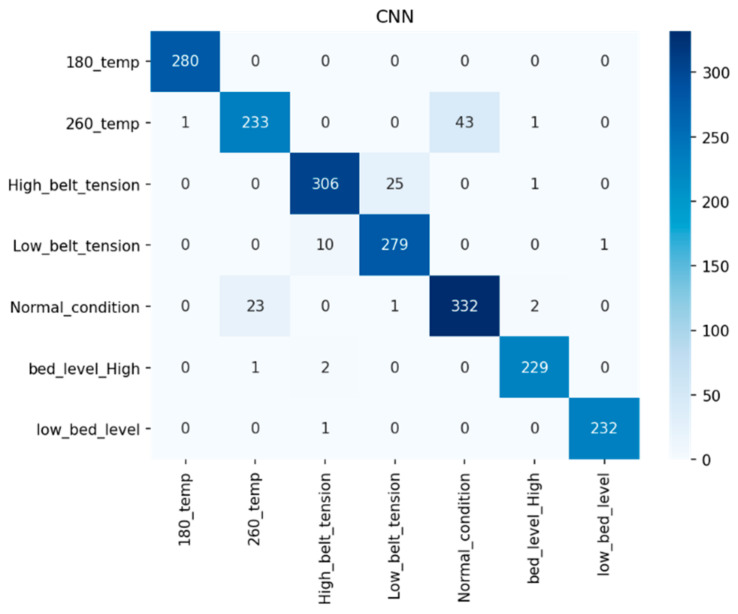
Multi-fault diagnosis using a confusion matrix.

**Figure 13:**
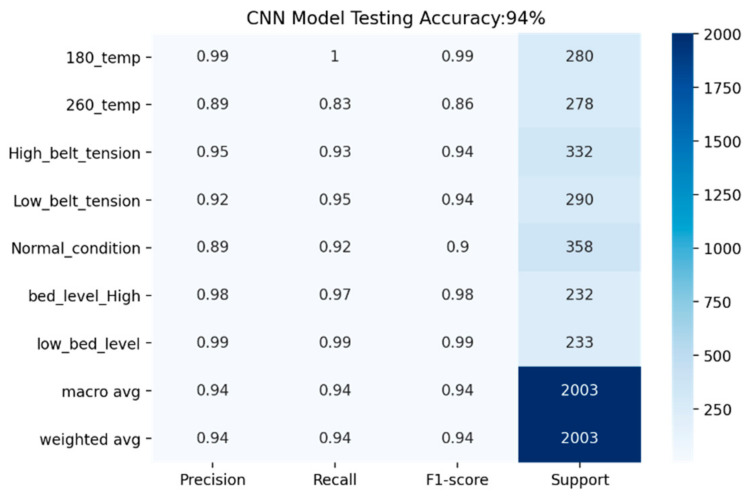
Performance evaluation using the CNN model.

The performance of the CNN model is evaluated using different performance evaluation parameters. Figure 12 shows that multiple fault classification is performed using the CNN model by referring to the confusion matrix and calculates precision, recall, and F1-score for an individual class, as shown in Figure 13.

### 4.6. Anomaly Detection Using K-Means Clustering Algorithm

**Figure 14:**
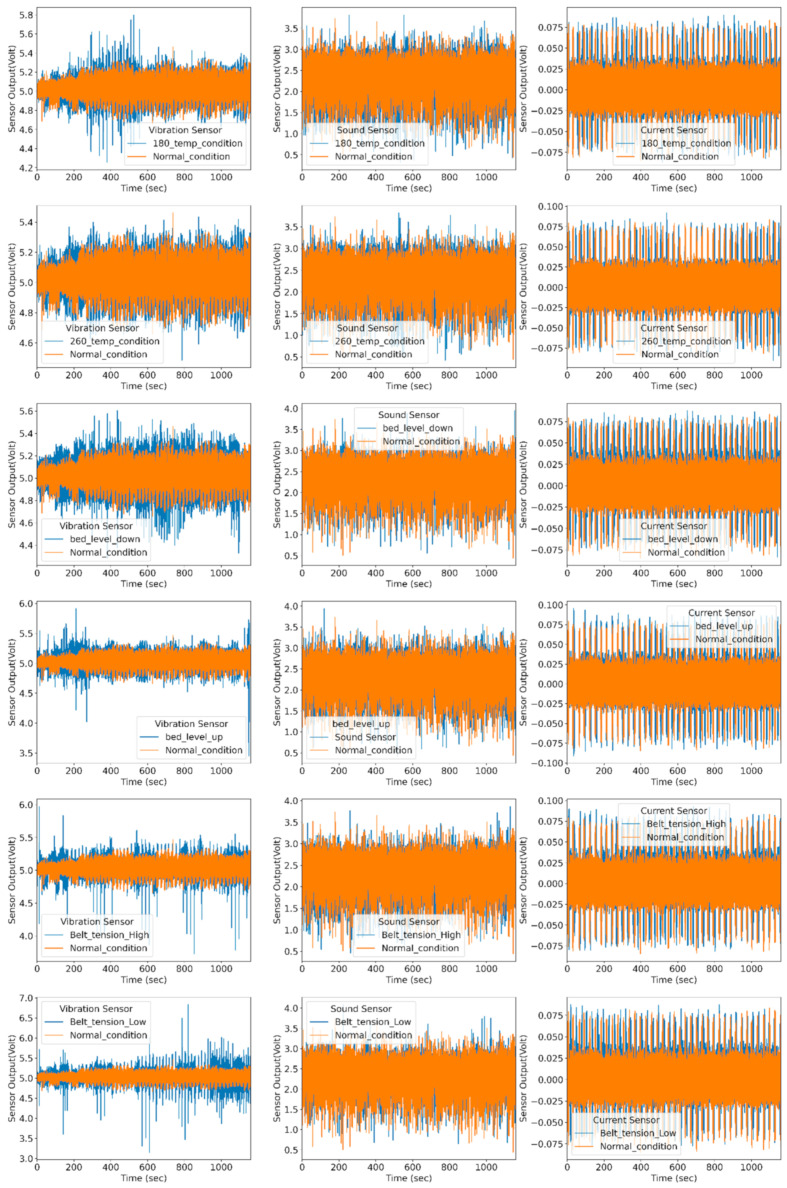
Representation of normal and abnormal condition vibration, sound, and current sensor data.

K-means is an unsupervised learning algorithm that deals with unlabeled data and groups them into several clusters. Each cluster groups similarity of data and shows dissimilarity with the remaining cluster. It assigns data points to clusters, such as a sum of the square distance between the cluster centroid and the data point should be minimum. The minimum distance shows that the defining cluster has homogeneous data points and well-separated distance with individual clusters. Since it refers to a distance-based mechanism to group similarities in the data, it needs to be fed standardized data, which have zero mean and one standard deviation. Therefore, the actual data are normalized using min–max, standardization, or z-score-based scaling methods. Figure 14 represents the normal and abnormal condition vibration, sound, and current sensor data. The normal and faulty printed product data are used for comparison to find the anomalies from the data. The authors performed exploratory data analysis on vibration, current, and sound sensor signals to detect anomalies. The *X*-axis of the subplot represents time in seconds, whereas the *Y*-axis of each subplot indicates sensor output in voltage.

**Figure 15:**
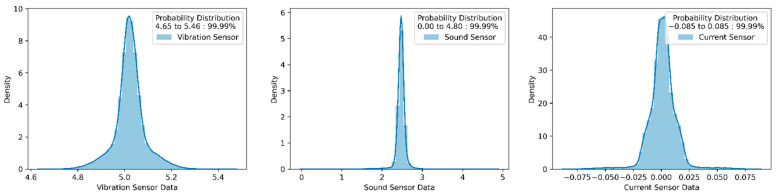
Vibration signal bell curve for deciding threshold values (for normal conditions data).

Each subplot of Figure 14 is compared with the normal condition to identify anomalies. For anomaly detection, the proposed k-means algorithm is used. The k-means algorithm divides the dataset into two groups based on the distancing mechanism. Each cluster has multiple sensors signals plot the normal distribution curve that represents the probability of data distribution symmetrically on both sides, as shown in Figure 15. Data points away from the bell curve represent the distribution of data in terms of standard deviations. A low standard deviation indicates that data are clustered toward the mean or center, showing higher normal deviation density. However, the large standard deviation indicates that data spread away from the mean and generate a wide and flatter curve. The probability of data distribution is calculated using Equation (11):

(11) $$f\left(x\right)=\frac{1}{\sigma \sqrt{2\pi }}e^{\frac{{\left(x-\mu \right)}^{2}}{2\sigma^{2}}}$$

where *f*(*x*) is a probability distribution, σ is standard deviation, σ^2^ is variance, *x* is the variable, and μ is the mean value of a variable.

**Figure 16:**
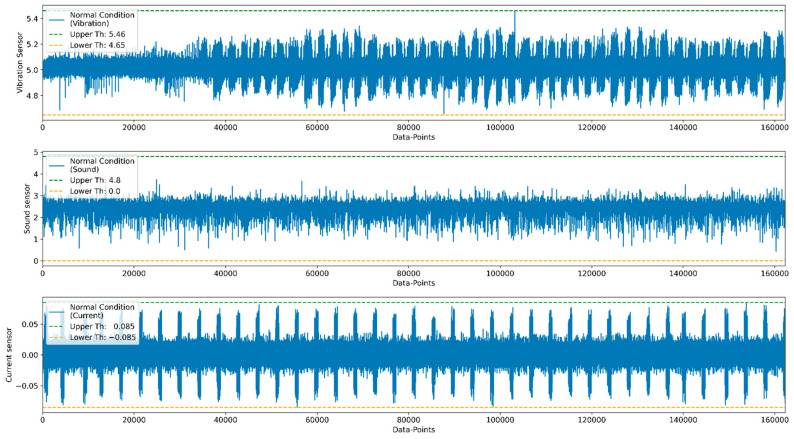
Threshold selection for normal data.

The point that covers 99.99% of the data distribution is selected from the bell curve as a threshold value for fault conditions, as shown in Figure 16. The values that cover 99.99% of the distribution from each cluster are set as an upper and lower threshold value for normal conditions. Using the same methods, the threshold value for multiple sensors is calculated. These upper and lower limits act as a boundary condition for remaining sensors, and based on it, anomalies in each condition can be calculated.

**Figure 17:**
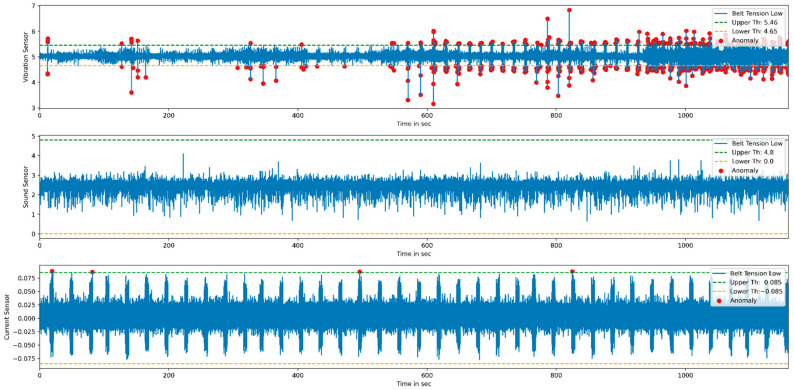
Anomalies detection for low belt tension.

**Figure 18:**
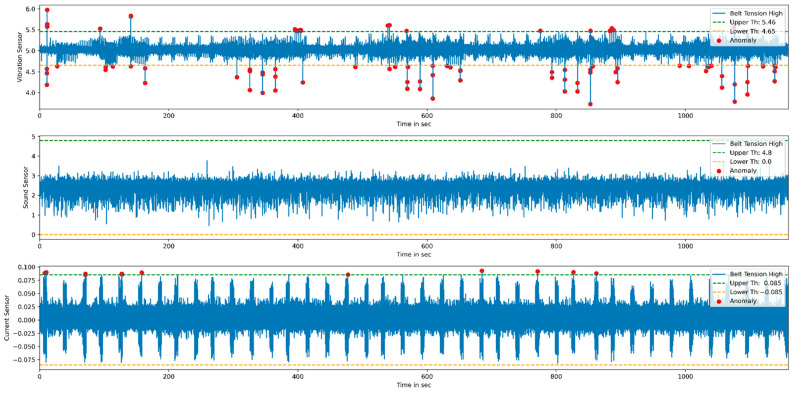
Anomalies detection for high belt tension.

For the anomaly detection purpose, multiple sensor data such as vibration, current, and sound are considered. The threshold values are calculated from the normal condition that divides the data into normal and anomaly data. Figure 17 and Figure 18 represent anomaly detection for low and high belt tension conditions. Once the threshold values are finalized, the real-time status of the product during the printing stage can be monitored by using the developed GUI.

## 5. Conclusions

In this work, a low-cost data acquisition system (DAQ) is designed using an Arduino micro-controller that captures real-time multi-sensor signals using various sensors such as vibration, current, and sound. The important findings of the study are quickly summarized as follows:

1. The multiple sensor’s real-time data was captured through a developed low-cost DAQ system with a uniform sampling rate of 140 samples per second (Hz) using an Arduino microcontroller board.
2. Multiple faults are induced in the 3D product, and the corresponding variations in the multiple sensor signals are recorded.
3. Time and frequency domain analysis is performed, and features are selected using the chi-square method.
4. The k-means cluster algorithm is used for data clustering purposes, and a bell curve or normal distribution curve is used for defining the individual sensor threshold values under normal conditions.
5. The CNN model is used to classify the normal and faulty data to evaluate the model’s performance based on recall, precision, and F1 score. Around 94% classification accuracy is obtained by using the CNN model, and corresponding anomalies from individual sensors are also determined.

## Figures and Tables

**Table 2:** Sampling frequency of individual and combined sensors.

Sensors	Sampling Frequency (Hz)
Sound	1537–1540
Current	365–370
Vibration (along X, Y, Z direction)	206–210
Sound + Current	328–300
Current + Vibration	150–155
Sound + Vibration	195–200
Current + Vibration + Sound	140

**Table 3:** Time domain and frequency domain features.

Feature	Feature Name	Formula
Time domain	Root Mean Square	$Y_{rms}=\sqrt{\frac{1}{N}\;\overset{N}{\underset{i=1}{\sum }}y_{i}{}^{2}}$
	Mean	$Y_{rms}=\frac{1}{N}\;\overset{N}{\underset{i=1}{\sum }}y_{i}$
	Variance	$y_{var}=\overset{n}{\underset{i=1}{\sum }}{\frac{\left(y_{i}-y_{m}\right)}{\left(N-1\right)}}^{2}$
	Skewness	$Y_{skew\;}=\frac{\;\sum_{i=1}^{N}{\left(y_{i}-y_{m}\right)}^{3}}{\left(N-1\right)y_{\sigma }{}^{3}}$
	Kurtosis	$Y_{kur\;}=\frac{\;\sum_{i=1}^{N}{\left(y_{i}-y_{m}\right)}^{4}}{\left(N-1\right)Y_{\sigma }{}^{4}}$
	Standard Deviation	$Y_{std}=\sqrt{\overset{n}{\underset{i=1}{\sum }}{\frac{\left(y_{i}-y_{m}\right)}{\left(N-1\right)}}^{2}}$
	Shape Factor	$Y_{SF}=\frac{\sqrt{\frac{1}{N}\;\sum_{i=1}^{N}y_{i}{}^{2}}}{\frac{1}{N}\;\sum_{i=1}^{N}y_{i}}$
	Clearance Factor	$Y_{cf}=\frac{max\left(y_{i}\right)}{\frac{1}{N}\;\sum_{i=1}^{N}y_{i}{}^{2}}$
	Peak to Peak	$Y_{peak\;}=max\left(y_{i}\right)-min\left(y_{i}\right)$
	Crest Factor	$Y_{cf}=\frac{Y_{max}}{Y_{rms}}$
	Impulse factor	$Y_{if}=\frac{Y_{max}}{Y_{peak}}$
Frequency domain	Spectral Mean	$F_{rms}=\frac{1}{N}\;\overset{N}{\underset{i=1}{\sum }}f_{i}$
	Spectral Variance	$F_{var}=\overset{n}{\underset{i=1}{\sum }}{\frac{\left(f_{i}-f_{m}\right)}{\left(N-1\right)}}^{2}$
	Spectral Standard Deviation	$F_{std}=\sqrt{\overset{n}{\underset{i=1}{\sum }}{\frac{\left(f_{i}-f_{m}\right)}{\left(N-1\right)}}^{2}}$
	Spectral Skewness	$F_{skew\;}=\frac{\;\sum_{i=1}^{N}{\left(f_{i}-f_{m}\right)}^{3}}{\left(N-1\right)f_{\sigma }{}^{3}}$
	Spectrum Kurtosis	$f_{kur}=\overset{N}{\underset{i=1}{\sum }}{\left(\frac{\left(f_{i}-f_{m}\right)}{\sigma }\right)}^{4}\;S\left(f_{i}\right)$

**Table 4:** Selected features by the chi-square method.

Sr. No	Feature	Chi-Square Score	Sr. No	Feature	Chi-Square Score
1	powspc_kurtosis_current	749.0191	21	IF_vib3	54.45829
2	powspc_skew_current	541.3756	22	MF_vib3	54.41491
3	mean_current	376.5628	23	CF_vib3	54.41491
4	kurtosis_vib3	255.8377	24	powspc_mean_current	54.4077
5	kurtosis_vib2	230.5496	25	std_vib1	53.96427
6	mean_vib2	139.974	26	rms_current	46.5927
7	rms_vib2	136.3834	27	var_vib1	44.64349
8	mean_vib3	114.8013	28	SF_vib1	44.49147
9	powspc_p2p_current	114.0053	29	std_sound1	43.93499
10	rms_vib3	113.5372	30	mean_vib1	43.30074
11	p2p_vib2	92.40597	31	rms_vib1	42.90891
12	peak_vib2	92.40597	32	var_vib3	41.39019
13	powspc_std_current	81.00898	33	SF_vib3	41.35895
14	IF_vib2	77.83489	34	p2p_vib1	38.15497
15	CF_vib2	77.71817	35	peak_vib1	38.15497
16	MF_vib2	77.71817	36	std_vib3	34.05571
17	powspc_rms_current	75.84044	37	SF_vib2	31.32263
18	kurtosis_vib1	69.38061	38	var_vib2	31.23275
19	p2p_vib3	65.75025	39	SF_sound1	30.55542
20	peak_vib3	65.75025	40	var_sound1	30.53649

**Figure A1:**
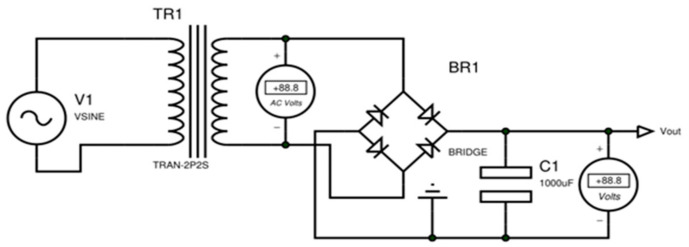
Full Wave Bridge Rectifier Circuit.

**Figure A2:**
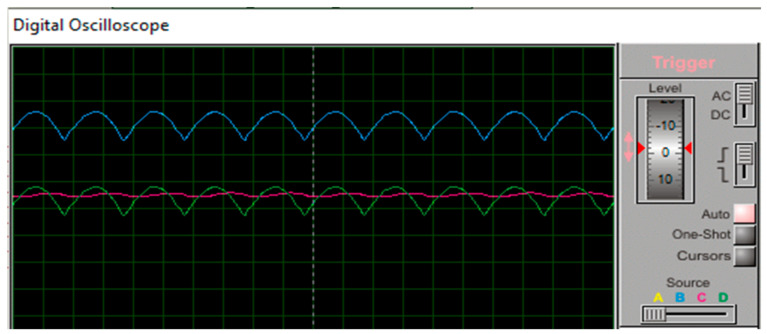
Waveform of Rectifier Circuit.

**Figure A3:**
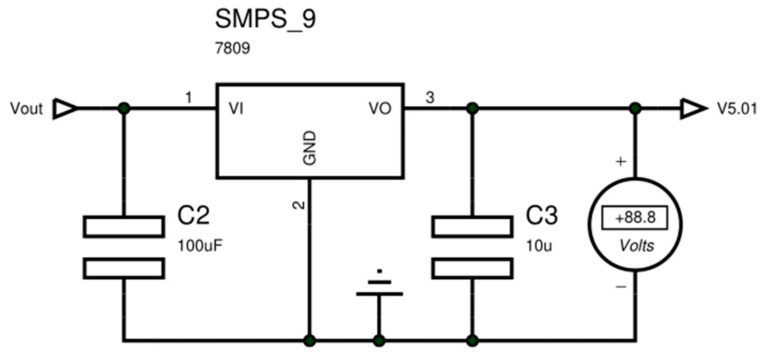
Voltage regulation using 7809.

**Figure A4:**
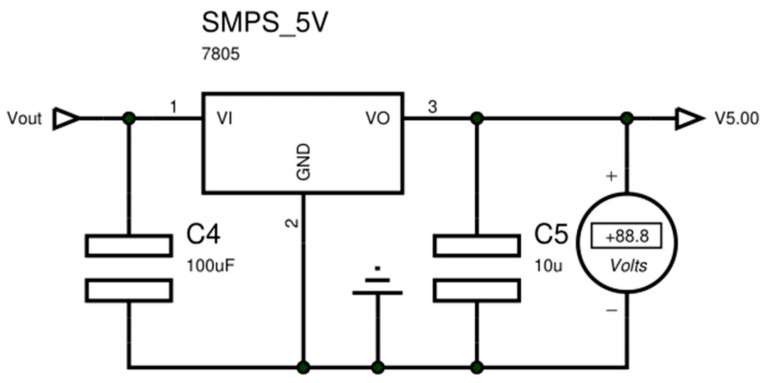
Voltage regulation using 7805.

**Figure A5:**
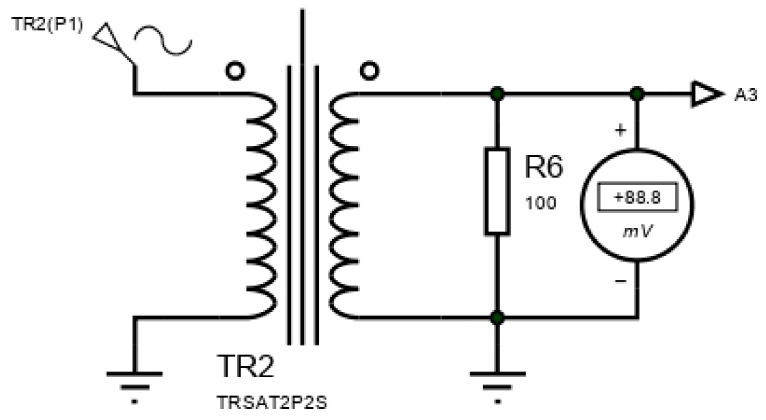
Current transformer.

**Figure A6:**
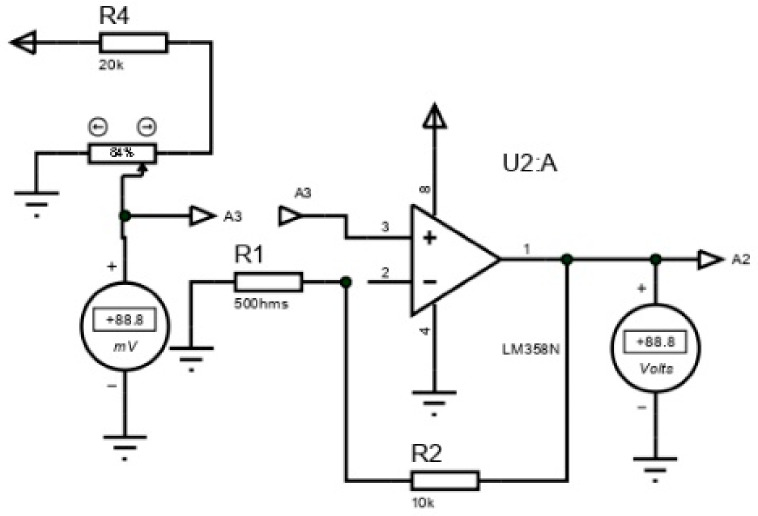
Operational amplifier.

## Data Availability

Not applicable.

## Abbreviations

ABS	Acrylonitrile Butadiene Styrene
AM	Additive Manufacturing
ADC	Analog to digital converter
CSV	Comma-Separated Values
CNN	Convolutional Neural Network
CFM	Cubic Feet per Minute
CT	Current transformer
DAQ	Data Acquisition System
DLP	Digital Light Processing
DC	Direct Current
FFT	Fast Fourier Transform
FBG	Fiber Bragg Grating
FOS	Factor of Safety
FDM	Fused Deposition Modelling
GUI	Graphical User Interface
IC	Integrated Circuit
I2C	Inter-Integrated Circuit, eye-squared-Communication
MEMS	Micro-Electro-Mechanical System
PLA	Polylactic Acid
PSD	Power Spectrum Density
PCB	Printed Circuit Board
SLS	Selective Laser Sintering
SPI	Serial Peripheral Interface
SRAM	Static random-access memory
SLA	Stereolithography
SCADA	Supervisory Control and Data Acquisition
SMPS	Switch Mode Power Supply
USB	Universal Serial Bus

## Appendix A

### Appendix A.1. Arduino Uno Specification

The proper selection of data acquisition is an essential aspect of any data monitoring system. Due to its high cost, acquiring experimental equipment has become a challenge. The Arduino board is used to partly solve this problem because it is low-cost and easy to practical equipment control [48]. The Arduino Uno is the microcontroller with an internal chip of atmega328p that operates at 16 MHz clock speed with 32 kb Flash memory and 2 kb Static random-access memory (static RAM or SRAM), the specification of the Arduino Uno board is shown in Table A1. It has digital and analog input–output pins that use to connect different sensors to the microcontroller. Those input–output pins designation can be changed through Arduino software. The Arduino board can be powered by a Universal Serial Bus (USB) cable or external power supply that varies from 6 to 20 volts. It has digital and analog input–output pins that can connect to several boards and circuits to perform various control and monitoring operations. It has an inbuilt analog to digital converter that measures analog sensor output through 6 pins and digital sensor output through 14 pins. It also incorporated with digital and analog output given sensors using I2C, Serial Peripheral Interface (SPI) communication interfaces [49]. In this proposed work, vibration and current sensors communicate with Arduino using RS485 and I2C protocols. However, the sound sensor output was directly given to the Arduino analog to digital converter to measure the sound intensity. Real-time data can be monitored through the serial monitor at a baud rate of 115,200 bps. It sends real-time sensors data to Python IDE using serial communication.

**Table A1:** Specification of Arduino Uno.

Parameter	Specification
Clock speed	16 MHz
Operating voltage	5 V
Analog Pins	6
Digital Pins	14
SRAM	2 kb
Flash Memory	32 kb

### Appendix A.2. Power Supply

All electronic devices are designed to work at a pre-define power rating based on voltage and current. Generally, the voltage supply to an electronic device is fixed or ideally constant, and current consumption is changes due to changing load conditions. The voltage regulation is one of the essential parameters of power supply modules that affect the performance of the complete circuits. Hence constant power supply is essential for the smooth working of DAQ.

The requirement for a consistent voltage supply is met in this proposed DAQ system by employing design voltage regulating circuits for 5 V, 9 V, and for a 24 V proposed switch mode power supply (SMPS) is used. The VBR1 is the industrial sensor that requires a 24 V supply as input and gives output that varies from 4 to 24 mA and 0 to 10 V using an analog and RS485 terminal. For the remaining sensors such as sound and current, a 5 V power supply is required. The external cooling fan is connected to a circuit that cools voltage regulating IC 7809 and 7805 by connecting to a 9 V power supply.

### Appendix A.3. Sound Sensor

It is a sound detection module that uses a capacitive microphone and inbuild Max4466 adjustable gain amplifier to measure sound waves through their intensity and converters them into an electrical signal. It is also used to measure and record changing voice or audio that play an important role in further calculating FFT from the sound signal. It uses a capacitive microphone soldered in a module that has a 20 kHz operating frequency. Measuring audio waveform coming out from the output pin of the module has VCC/2 DC bias, so at the perfect quiet place, it was given steady output, which is half of the supplied voltage (VCC/2). It also provides AC-coupled audio by connecting a 100 μF capacitor between the sensor’s output pin and the analog input pin of the Arduino board. The output getting from the module is not suitable for driving speakers directly. For that purposed external audio amplifier is required. In this proposed system, the Max4466 Sound sensor given output is directly fed to the analog terminal of the Arduino Uno board. As per changing the sound intensity, the output voltage given by the sound sensor is increased up to 5 V. The specification of the sound sensor considered during selection is described in Table A2.

**Table A2:** Specification of the sound sensor.

Parameter	Specification
Model	MAX4466 Electret Microphone Amplifier
Supply Voltage	2.41 to 5.5 V
Power-Supply Rejection Ratio	112 dB
Common-Mode Rejection Ratio	126 dB
Gain Bandwidth Product (kHz)	600

### Appendix A.4. Current Sensor

The current transformer is an electrical device that is used to measure alternating current. It is mostly used to determine the amount of electricity consumed by electronic equipment. It is a split core type device that can be clamped to the live or neutral wire of the electronic device to measure alternating current. It connects one wire at a time, either live or neutral wire. SCT-013-030 is a split-core current transformer having the jaw to clamp the wire. The current transformer is installed 90 degrees to the current-carrying conductor. A change in magnetic field induces a voltage in the secondary winding that is directly proportional to the amount of current flowing to the clamp wire, and its specification is described in Table A3.

**Table A3:** Specification of current sensor.

Parameters	Specification
Model	SCT-013-030
Input Current	0 to 30 A
Output	0 to 1 V
Frequency range	50 Hz to 1 kHz
Temperature range	−25 °C to 70 °C

### Appendix A.5. Vibration Sensor

VBR1/D0-3 is an industrial vibration sensor are operating on a 24 V power supply to give output that varies from 4 to 20 mA and 0 to 10 V. It has five terminals or wires. Out of five, two terminals are connected to a 24 V power supply, two-terminal are used for RS485 communication, and one terminal gives an analog output that varies from 4 to 20 mA. The same sensor data is captured through RS485 and analog wire, but at a time, one type of wiring connection is used to measure vibration. The VBR1/D0-3 is a Micro-Electro-Mechanical System (MEMS) sensor used to monitor and measure shocks, vibration, tilt, and temperature. Using Analog and RS485 terminal of vibration sensor measures resultant and individual axis vibration. This proposed system used an RS4855 terminal of a vibration sensor to measure the individual axis of vibration. Table A4 refers to describes the specification of vibration sensor that has a major contribution to sensor selection.

**Table A4:** Specification of Vibration Sensor.

Parameters	Specification
Model	VBR1/D0-3
Supply Voltage	24 V
Number of Axis	3
Frequency Range	400 Hz
Weight	100 g
Operating Range	±16 g

### Appendix A.6. ADS1015

It is an Analog to digital converter (ADC) device with a 12-bit resolution offered to the connected device. It is transfers data to the Arduino board using I2C communication. It has four I2C slave channels that address four devices at a time. It is powered by a single power supply that varies from 2.5 to 5.5 V. That also supports differential and single-end devices to measure the analog signal. ADS1015 is a device that has a sampling or conversion rate of 3300 samples per sec. It is suitable for an input range of ±250 mv and comfortable for large or small signals to be measured with High resolutions. It also has an inbuild multiplexer that gives two differential and four single-ended inputs. It is a programmable amplifier module that can switch its operating mode of channels through programming either single-end or differential ends. The ADS1015-Q1 device has two modes of operation: continuous conversion and single-shot conversion, which immediately powers down after a conversion and reduces current usage significantly during idle periods.

### Appendix A.7. RS485 Module

RS485 Module works on the asynchronous serial communication protocol [50], which means along with data, there is no synchronous clock pulse transmitted. It also used differential signals that transfer data from one device to another device using binary conversion. The differential signal is working on the differential voltage range ±5 V. One of the advantages of the RS485 module is that it rejects the common mode of noise. It transfers the data with a sampling rate of 30 MB per second. It also has a provision to support multiple slave devices using a single master mode. It can connect with 32 devices at a time. In this proposed work, the RS485 module communicates with the vibration sensor and transmits sensor data to the Arduino board. RS485 module can be used as a transmitter as well as the receiver. In the case of the transmitter, receiver Enable (RE), and Driver Enable (DE) pin of module connected to 5 V supply and DI pin of the module is connected to transmitter pin of Arduino, then data from transmitting (Tx) pin of the Arduino was sent to DI pin of the module. In the case of the receiver, the RE and DE pins of the module are connected to the ground. Then, from the RO pin of the RS485 models, send the complete sensor data to the Arduino board.

## Appendix B

### Appendix B.1. Power Supply Unit

#### The Full-Wave Bridge Rectifier Circuit

In a bridge rectifier, both half cycle of the sine wave is rectified by connecting four diodes together to form a bridge circuit. The secondary winding of the transformer is generally connected to the bridge circuit on one side and another side connected with a load, as shown in Figure A1. It converts AC output voltage into pulse Direct Current (DC) output voltage that can further be used as a power supply after smoothing output voltage using a capacitor. The advantage of a full-wave bridge circuit is that it does not require a center-tapped transformer that reduces the cost and size of the circuit. It is also suitable for a single secondary winding that is directly connected to the bridge and provides constant voltage across load or resistance. Here current flowing through the load is unidirectional, so voltage develops across load should be the same.

The average DC voltage across the load is calculated by Equation (A1).

(A1) $$V_{avg}=0.637\;V_{peak}$$

In actual implementation, the voltage getting across the load is not meeting with simulation output. Because of the diode, it does not turn on until the source voltage reaches 0.7 V. The bridge circuit operated on two diodes, so 1.4 V source voltage is lost due to the diode.

The corrected peak output voltage getting from the full-wave bridge rectifier circuit is calculated by Equation (A2).

(A2) $$V_{avg}=0.637\;\left(V_{peak}-1.4v\right)$$

The full-wave bridge rectifier inverts each negative cycle and doubles the number of positive cycles. Therefore, it gives an output frequency that is twice than input as per Equation (A3).

(A3) $$f_{out}=2f_{\begin{array}{c} in\; \end{array}}$$

The output voltage from the rectifier circuit is in pulse form that increases up to peak voltage and decreases until zero volts. Such output cannot be used as a power supply. The minimum fluctuation or ripper is required to get a steady or constant DC power supply from the circuit.

The maximum ripper voltage getting from the full-wave bridge rectifier is calculated by Equation (A4).

(A4) $$V_{ripper}=\frac{I_{load}}{f*C}$$

A filtered signal need to be received from a full-wave bridge circuit to obtain constant voltage. This problem can be solved by connecting a 1000 μF capacitor to a bridge circuit. The connected capacitor charge itself till the peak voltage, then it discharges itself to maintain the constant output voltage, as shown in Figure A2.

Root Mean Square Error value of an output voltage of full-wave bridge rectifier calculated by Equations (A5) and (A6).

(A5) $$V_{rms}=\frac{V_{peak}}{\sqrt{2}}$$

(A6) $$V_{avg}=0.9\;V_{rms}$$

Crest or Peak Factor of the output voltage of full-wave bridge rectifier circuit calculated by Equations (A7) and (A8) represents peak to peak voltage getting as output.

(A7) $$V_{crest}=\frac{V_{peak}}{V_{rms}}$$

(A8) $$V_{peak-to-peak}=2V_{peak}$$

The form factor of the output voltage of the full-wave bridge rectifier circuit is calculated by Equation (A9).

(A9) $$V_{f}=\frac{V_{rms}}{V_{avg}}$$

The 7805, 7809, 7812, 7824 IC are a member of the 78xx series of voltage Regulation families. It is mainly used for giving a steady or constant voltage. Figure A3 and Figure A4 represent the actual voltage regulating circuit used in the proposed system. In Figure A3, the output taken from the full-wave rectifier circuit is given as input to 7809 IC, and at the output, it provides 10 V as a power supply. The 7809 IC has three terminals; Inputs, Ground, and Output. The Input terminal of the IC is connected to the positive terminal of the 15.9 V supply. The negative terminal of the IC is connected to the ground terminal of the supply and gets a 10 V as output.

Using voltage regulation IC, high Input voltage converts into a low output voltage. Due to voltage differences across IC, a lot of energy is exhausted in the form of heat. When the difference between input and output voltage is high, then a large amount of heat is generated that affects the performance of voltage regulating IC. It’s required to dissipate generated heat for the voltage regulator IC as fast as possible for smooth functioning purposes. The heat dissipation problem can be solved in multiple ways like.

1. Selecting the appropriate size of the heatsink,
2. Keeping the low voltage difference across IC,
3. Using multiples voltage regulating IC that can convert high voltage supply into a lower supply voltage due to performing step by step reduction and cause a minimum voltage to drop across IC,
4. Selecting the appropriate cooling fan size, etc.

In this proposed system, the proper size of the cooling fan is selected to continuously passes air across IC with a mass flow rate of 60 Cubic Feet per Minute (CFM), and due to force convection, it dissipated generated heat to surrounding as fast as possible and keep IC surface temperature is low.

To calculate the amount of heat generated by the 7805 and 7809 voltage regulator IC, the following Equations (A10) and (A11) are referred.

(A10) $$Q_{5v}=\left(V_{in}-5\right)\;*\;I_{out}$$

(A11) $$Q_{9v}=\left(V_{in}-9\right)\;*\;I_{out}$$

The total amount of heat generated by the circuit $Q_{total}$ can be calculated by Equation (A12).

(A12) $$Q_{T}=\left(Q_{5v\;}+Q_{9v}\right)\;*\;FOS$$

Here,

$Q_{5v}$ = amount of heat generated by 7805 IC
$Q_{9v}$ = amount of heat generated by 7809 IC
*Q_T_* = Total amount of heat generated
$V_{in}$ = Input voltage given to voltage regulating IC
$I_{out}$ = output current given by output terminal of IC
*FOS* = factor of safety.

Those voltage regulating IC has provision for a heat sink that can be connected at the backside of the IC. Using the natural convection method, the cooling rate of IC is slow during load conditions. That’s one of the reasons which caused the failure of IC and power supply. In the force convection method, continuous air passes over the backside of the IC surface using an external fan that reduces IC temperature quickly and gives a smooth power supply.

The amount of heat dissipated by the cooling fan can be described by Equation (A13).

(A13) $$Q_{fan}=MC_{p}\left(T_{2}-T_{1}\right)$$

where

$Q_{fan}$ = Amount of Heat Generated by Fan
*M* = Mass flow rate in Kg/s
$C_{p}$ = Specific Heat of air
$\left(T_{2}-T_{1}\right)$ = Change in temperature.

The amount of air supply by Cooling Fan in CFM (cubic feet per minute) that can help to dissipate heat from voltage regulating IC is calculated by Equation (A14)

(A14) $$M_{CFM}=\left(1.76*\;Q_{T\;}\right)/\left(T_{2}-T_{1}\right)$$

To fulfill the requirements of experimental airflow supply by the cooling fan in CFM, an OD9225-12HHBIP68 cooling fan was selected, and their specifications are shown in Table A5.

**Table A5:** Parameters required for fan selection.

Part Name	Rated Voltage	Input Power	Rated Current	Rated Speed	Airflow (CFM)	Noise Level (dB)	Static Pressure
OD9225-12HHBIP68	12 VDC	3.0 W	0.29 A	3300 RPM	60	38	0.25″ H_2_O

### Appendix B.2. Sensor Configuration with DAQ System

#### Appendix B.2.1. Current Transformer (CT)

A current transformer is similar to a standard transformer with a primary, secondary winding, and magnetic core are as shown in Figure A5. The current flowing through the wire produces a magnetic field in the core that induces current into the secondary coil. The amount of current flowing through the measuring wire is directly proportional to the current flowing through the secondary winding.

The current flowing through secondary winding is calculated by Equations (A15) and (A16)

(A15) $$CT_{turns}=\frac{N_{primary}}{N_{secondary}}$$

(A16) $$I_{secondary}=CT_{turns}*\;I_{primary}$$

The output of the current transformer gets converted into voltage using a burden resistor. It completed a secondary winding circuit and was kept very low to maintain CT core saturations.

The primary peak and secondary peak current flowing through the winding of the current transformer is calculated by Equations (A17) and (A18).

(A17) $$I_{primary-peak\;}=\sqrt{2}\;I_{rms}$$

(A18) $$I_{secondary-peak}=\frac{I_{primary-peak\;}}{N_{s}}$$

The burden resistance connected to CT is calculated by Equation (A19) and is described below.

(A19) $$R_{burden}=\left(V_{ref}*\;CT_{turn}\right)/\left(2\sqrt{2}\;I_{max}\right)$$

In this proposed method, the non-inverting operational amplifier is connected to a current transformer that has the ability to convert the 1 mV output given by the current transformer into 19.5 mV. It also provides an amplified output signal that has the same phase as the input signal. It’s used negative feedback that is connected to the non-inverting terminal of the operational amplifier.

In Figure A6, R4 and variable resistance are connected together to give output that varies from 10 to 250 mV. Output is taken from variable resistance connected to DC voltmeter to the measured voltage difference across variable resistance. This voltage divider circuit is a replica of the voltage given by the current sensor. Equation (A20) calculates the voltage across variable resistance connected to the DC voltammeter and non-inverting terminal of the op-amp (operational amplifier).

(A20) $$V_{variable}=\frac{R_{variable}}{\left(R_{3}+R_{variable}\right)}\;V_{R3}$$

The input voltage signal given from the operational amplifier is gets amplified with a gain of 19.5 V. As input voltage changes with millivolt corresponding output voltage signal also change, which is measured with the help of a DC voltmeter. Equation (A21) represents the output voltage getting from the output terminal of the operational amplifier.

(A21) $$A_{gain}=\frac{V_{out}}{V_{in}}=\left(1+\frac{R_{2}}{R_{1}}\right)$$

In Figure A6, the circuit used external resistance R1 and feedback resistance *R*_2_ that connects to inverting terminal of the operational amplifier, and by applying Kirchhoff’s law, the voltage across *R*_2_ resistance is calculated by Equation (A22).

(A22) $$\frac{V_{in}}{R_{1}}=\frac{V_{out}-V_{in}}{R_{2}}$$

Here, the input voltage applied to the non-inverting terminal of an op-amp is *V_in_*_,_ and the output voltage getting from op-amp is *V_out_*. Equation (A23) calculates the gain of the closed-loop circuit

(A23) $$V_{out}=\left(1+\frac{R_{2}}{R_{1}}\;\right)V_{in}$$

However, in an actual implementation as compared to the designed operational amplifier, the ADS1015 board gives better signal amplification. This board uses I2C communication protocols and is suitable for measuring readings from differential and single-ended devices.

#### Appendix B.2.2. Sound Sensor

The sound sensor gives differential output that can vary from 2.5 to 5 volts. The differential voltage getting from the sound sensor as output is input for Arduino analog pins. This output voltage is divided into 204 steps to convert it into analog form. This analog output range varies from 0 to 1023 steps and is mapped with its measuring unit of the maximum and minimum value in general. To calculate analog value given by sensor Equations (A24) and (A25) are referred.

(A24) $$V_{in}=\frac{A_{in}*5}{1023}$$

(A25) $$A_{in}=\frac{V_{in}*1023\;}{5}$$

The intensity of sound measured by the sensor is calculated by Equation (A26).

(A26) $$db_{sound}=20*log\left(\frac{A_{in}}{A_{ref}}\right)$$

Here,

$db_{sound}$ = Measured Intensity of sound in dB
$A_{in}\;$= The analog value generated by applying a differential voltage to the sound sensor as output
$A_{ref\;}$ = The analog value generated by applying reference voltage (VCC/2).

#### Appendix B.2.3. Vibration Sensor

The vibration sensor gives output that varies from ±16 g. When the vibration sensor is connected to the Arduino board through the RS485 module [51], it sends raw vibration data that is further converted in decimal after multiplying the correction factor, as shown in Equation (A27).

(A27) $$g_{vibration}=A_{raw\_analog}*C_{correction\_factor}$$

The correction factor for raw vibration is given in Table A6.

**Table A6:** Correlation Factor for Raw Vibration Sensor.

Full Scale in g	Correction Factor
2	6.1037 × 10^−5^
4	1.2207 × 10^−4^
8	2.4415 × 10^−4^
16	4.88305 × 10^−4^
